# The Care Coordination Program: a virtually integrated care delivery model for complex, high-needs patients

**DOI:** 10.1186/1472-6963-11-S1-A18

**Published:** 2011-10-19

**Authors:** R Moodley Naidoo, L Steenkamp

**Affiliations:** 1 Risk and Quality Management, Discovery Health, Sandton, 2146, South Africa

## Background

The South African healthcare sector is fragmented.

The increasing prominence of non-communicable, or chronic, diseases in both the private and public healthcare sectors contributes to the country’s complex and costly “quadruple burden of disease” (the others being HIV/AIDS, tuberculosis, maternal and child mortality, and violence). These chronic diseases, once they have progressed, are inherently difficult to manage due to their underlying psychosocial components and their characteristically complex co-morbidities. High costs, without the desired improved clinical outcomes, are common.

Elsewhere in the world, such as in the United States, where similar fragmented systems exist, new care-delivery models have emerged to better manage patients with complex conditions. These include vertical payer-provider integration, patient-centered medical homes, and accountable care organizations. Enablers of similar structural reforms are lacking in South Africa, however, where regulatory rules bar the employment of salaried doctors by hospital networks. Moreover, a looming nation-wide dearth of healthcare professionals diminishes the potential for systemic structural changes to the status quo.

Discovery Health (DH) is the country’s largest private healthcare payer, providing health insurance coverage to over 2.5 million people. The Care Co-ordination Program (CCP) is DH’s response to the fragmented care received by its members who present with complex healthcare needs, including psychological and social vulnerabilities.

## Methods

**Figure 1:**
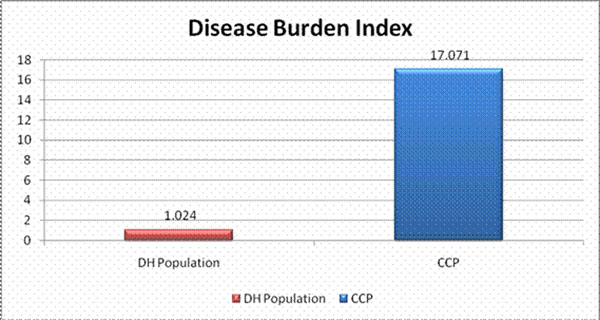
Disease Burden Index for CCP members compared to the rest of the DH population.

The target DH population of members likely to benefit in the CCP is identified geographically using the Johns Hopkins Adjusted Clinical Group tool. This tool categorizes members into Resource Utilization Bands. Further, a Disease Burden Index (DBI) is employed to narrow the focus of the CCP to DH members with the greatest complexity and highest disease burden. The DBI for the CCP population is 17.071 compared to a significantly lower DBI for the general DH population of 1.024 (see Figure 1).

Sub-acute service providers in the identified high-needs geographic areas who meet structural, service and management criteria are contracted with DH to participate in a CCP network.

At the time of a patient’s admission into an acute facility, a DH care recruiter employs pre-set clinical, social, and psychological entry criteria combined with a FIM (Functional Independence Measure) score to identify patients at risk of sub-optimal quality of care associated with repeated costly admissions. The identified patients are voluntarily enrolled in the CCP and, at this point, a DH care co-coordinator joins the care team of the contracted service provider. The care co-coordinator ensures that the patient’s unique needs are carefully and methodically addressed by the service provider’s interdisciplinary care team.

Integrating the family into the care plan is central to ensuring a successful transition to the home. The care co-coordinator shares the transition plan and the patient’s electronic medical record with all involved providers, thus ensuring the co-ordination of care following discharge. The co-coordinator is a valuable resource for the patient, managing vulnerabilities during the transition to home and community.

The CCP discharge goal is an empowered patient reintegrated into a safe physical environment supported by knowledgeable caregivers.

## Results

Improved clinical outcomes and cost efficiencies are evident from the CCP.

**Figure 2:**
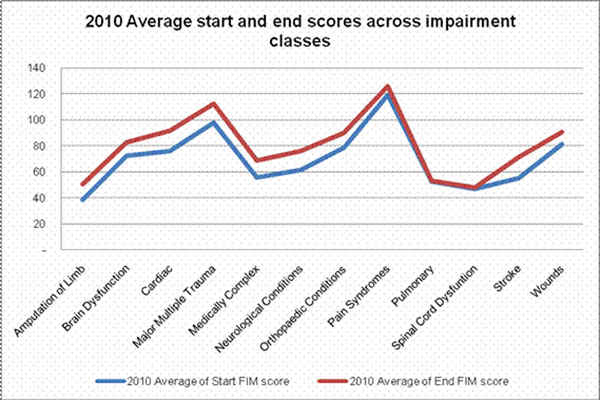
2010 Average FIM score gains across clinical impairment class.

In 2010, an average increase of 19% in the FIM score was observed in CCP patients from admission to discharge. Patients with FIM scores between 30 and 80 had the highest FIM gains – an average increase of 27%. Across clinical impairment classes, CCP patients with neurological conditions, stroke, and cardiac illness had the highest average FIM gains (see Figure 2).

In 2010, the average monthly cost for the 175 members who participated in the CCP decreased from 23,307 SAR (South African Rands) pre-CCP enrollment to 8,672 SAR following CCP enrollment – a reduction of 62.8%.

## Conclusion

The CCP continues to gain traction with 575 DH members currently enrolled in the program. Results thus far are striking, and they justify a greater national presence for the program, as well as its underlying principles of co-ordination and integration across traditional structures. The analytic capabilities and tools employed in the selection and management of the relatively small, current CCP population remain to be tested in a larger national CCP network.

